# Attentional bias modification for chocolate: Sham-n training as a new control group

**DOI:** 10.1371/journal.pone.0260294

**Published:** 2021-11-19

**Authors:** Eva Kemps, Marika Tiggemann

**Affiliations:** School of Psychology, Flinders University, Adelaide, Australia; Universidad Autonoma de Madrid, SPAIN

## Abstract

Although attentional bias modification has been shown effective in several appetitive domains, results have been mixed. A major contributor seems to be the choice of control condition. The aim of the present study was to compare attentional bias modification for chocolate against a new control condition, sham-n (neutral or no-contingency) training. Using a modified dot probe protocol, participants (N = 192; 17–30 years) were randomly trained to attend to chocolate pictures, avoid chocolate pictures, or received sham-n training. In the attend and avoid conditions, stimulus pairs consisted of one chocolate and one non-chocolate picture, and probes replaced most often (90/10) chocolate or non-chocolate pictures, respectively. In the sham-n training condition, stimulus pairs consisted of two chocolate or two non-chocolate pictures, and probes replaced pictures within pairs with equal frequency (50/50). Attentional bias for chocolate increased following attend training, decreased following avoidance training, and did not change following sham-n training. The findings clearly demonstrate that both attend and avoidance training alter (in opposite direction) attentional bias for chocolate, whereas sham-n training is inert. This makes sham-n training particularly promising for use in clinical samples who tend to show strong initial biases.

## Introduction

Attentional bias refers to the tendency to selectively attend to certain stimuli over others. Although initially attentional biases were shown to exist in anxiety and depression [1, 2], they have now been demonstrated in appetitive domains, such as alcohol consumption, smoking and eating [3–5]. The most widely used protocol to measure attentional bias is the dot probe task [6]. In this task, pairs of stimuli (one self-relevant, one neutral) are presented briefly followed by a probe in the location of one of the stimuli, to which participants have to respond as quickly as possible. Probes replace the self-relevant and neutral stimuli equally often. Faster responses to probes that replace the self-relevant stimuli are taken as an indication of an attentional bias toward these stimuli [2].

MacLeod, Rutherford, Campbell, Ebsworthy and Holker [7] subsequently modified the dot probe task in order to reduce attentional bias. To this end, they varied the contingencies between stimulus and probe locations such that the probes most often replaced the neutral stimuli, thereby directing attention *away* from the self-relevant stimuli. They showed that training participants to redirect attention away from anxiety-related stimuli can reduce attentional bias for such stimuli. Attentional bias modification has since been applied to the appetitive domain with mixed results. While some studies have shown a reduction in attentional bias for substances such as alcohol, tobacco, drugs and chocolate [8–10], others have not [11, 12].

A number of methodological reasons have been put forward as possible explanations for the inconsistent results, including variations in sample composition, number of training sessions, study setting and mode of delivery across studies [13–18]. However, one major contributing factor, and the focus of the current paper, is the choice of control condition [19]. Some studies have compared attentional bias modification with a reverse training protocol (‘attend’) in which participants’ attention is directed *toward* substance cues. For example, Field and Eastwood [20] found that participants who were trained to direct their attention away from alcohol-related pictures (i.e., avoid) showed a reduced attentional bias for such pictures relative to participants who were trained to direct their attention toward them (i.e., attend). More recently, using a similar protocol, Kemps et al. [9] showed that avoidance training reduced attentional bias for chocolate pictures relative to attend training. Attentional bias modification studies for other substances, such as tobacco and drugs, have found the same pattern of results (e.g., [8]). However, one confounding issue is that the difference between the avoid and attend groups observed in these studies could result from a reduction in attentional bias for the avoid group, or an increase in attentional bias for the attend group, or both.

Other studies have instead used a different comparison condition known as sham training. Sham training is usually conceptualised as non-contingent because probes replace substance-related and neutral stimuli within dot probe pairs with equal frequency (50/50). For example, Schoenmakers et al. [10] compared avoidance training away from alcohol-related pictures with sham training. They found that attentional bias decreased following avoidance training relative to sham training. Likewise, Kerst and Waters [21] showed a reduction in attentional bias for smoking-related pictures relative to sham training. In the food domain, Zhang, Cui, Sun and Zhang [22] and Smith, Treffiletti, Bailey and Moustafa [23] similarly reported reductions in attentional bias for high-calorie food stimuli relative to sham training. However, in contrast, Hardman et al. [12] found no change in attentional bias for cake images following avoidance training relative to sham training. A similar lack in attentional bias change relative to sham training has been reported in bias modification studies on tobacco [11, 24].

Perhaps not surprisingly, studies that have used attend training as the control condition have generally found more consistent evidence for the effectiveness of attentional bias modification than those that have used sham training [15, 19]. Although sham training was designed not to manipulate attentional bias and is considered the gold standard control, there is growing evidence that it is in fact not inert [25, 26]. In particular, some attentional bias modification studies found no effect of avoidance training relative to sham training because both avoidance training and sham training were effective in reducing the bias [11, 12, 24]. In support, a recent meta-analysis of alcohol and tobacco studies concluded that there is a (small) reduction in bias following sham training [14]. Although sham training’s 50/50 contingency is usually conceptualised as non-contingent, it has been argued that it actively trains *equal* attention to substance-related and neutral stimuli [27]. Logically, then, the stronger an individual’s initial level of attentional bias, the greater should be the effect of sham training. This logic makes sense of the observation that sham training tends to show a greater reduction in attentional bias in clinical samples than in samples of convenience [15, 19].

In response to the growing concerns about sham training, Tiggemann and Kemps [27] recently proposed an alternative control protocol, called sham-n (neutral or no-contingency) training. Sham-n training resembles sham training in that it matches exactly the stimulus exposure and response requirements of attentional bias modification. Participants still see the same stimulus pictures and respond to the probes in exactly the same way (i.e., identify the location of the probe). However, sham-n training has stimulus pairs that consist of two substance-related stimuli or two neutral stimuli rather than one substance-related stimulus and one neutral stimulus. Stimulus and probe locations are never paired with one another (unlike the 50/50 contingency in sham training), and thus there is truly no contingency. Consequently, sham-n training (unlike sham training) should have no effect on any pre-existing attentional bias.

The aim of the present study was to compare the effectiveness of avoidance training against both attend training and sham-n training on attentional bias for chocolate. In so doing, we sought to determine whether the difference in bias observed in previous studies (e.g., [8, 9, 20]) is driven by the attend or avoidance training or both.

## Materials and methods

### Participants

Participants were 192 female undergraduate students at Flinders University aged 17 to 30 years (*M* = 20.86, *SD* = 2.85). The sample size was based on a power analysis to detect a moderate-sized effect with an alpha level of.05 and 80% power [28]. Participants received course credit or an honorarium in lieu of their time and commitment. As hunger has been linked to attentional biases for food [29], participants were instructed to eat something two hours before the testing session to ensure they were not hungry. All participants reported having complied with this instruction. Additionally, participants rated their level of hunger on a 100-mm visual analogue scale, ranging from “not hungry at all” to “extremely hungry”. Hunger ratings were below the mid-point of the scale (*M* = 45.37, *SD* = 24.05), and importantly, did not correlate with attentional bias scores at pre-test, *r* = .05, *p* = .465, or post-test, *r* = -.07, *p* = .334. Furthermore, all participants reported that they liked chocolate, in response to the yes/no question “Do you like chocolate?”

### Study design

The experiment employed a 3 (training condition: attend, avoid, sham-n) × 2 (time: pre-test, post-test) between-within subjects design. Participants were randomly assigned to the training conditions, subject to equal numbers per condition.

### Stimuli

The stimuli for the modified dot probe task were constructed from 80 digital coloured photographs comprising 20 pictures of chocolate or chocolate-containing food items (e.g., brownie, chocolate mousse) and 60 pictures of highly desired food items not containing chocolate (e.g., cake, pizza), derived from Kemps, Tiggemann and Elford [30]. Pilot data showed that the target (chocolate) and control (non-chocolate) stimuli were well-matched on overall appeal, in contrast to, for example, household items or office supplies which are often used as comparison control stimuli in dot probe studies (e.g., [31, 32]). All pictures were scaled to 120mm in width, whilst maintaining the original aspect ratio. Three sets of 20 stimulus pairs were constructed: 20 critical (chocolate–non-chocolate sweet food), 20 control (non-chocolate–non-chocolate), and 20 unpaired (10 chocolate–chocolate, plus 10 non-chocolate sweet food–non-chocolate sweet food). Another 14 picture pairs, with no food related content (e.g., car, beach ball) were taken from Kemps et al. [9], and used for practice and buffer trials.

### Procedure

The study protocol was approved by the Flinders University Social and Behavioural Research Ethics Committee. Participants were tested in a quiet room in the Applied Cognitive Laboratory in a single session of 30 min. duration. Participants were seated at a desk, approximately 50 cm in front of an IBM-compatible computer with a 22-inch monitor. After giving written informed consent, participants completed a brief demographics questionnaire, which included the hunger measure, followed by the modified dot probe task.

Following standard attentional bias modification protocols [20], the modified dot probe procedure consisted of three phases: (1) a pre-training baseline assessment of participants’ attentional bias for chocolate (pre-test), (2) a training phase in which a third of participants were trained to attend to chocolate, a third were trained to avoid chocolate, and a third received sham-n training, (3) a post-training assessment of participants’ attentional bias for chocolate similar to the pre-test (post-test).

#### Pre-test

At pre-test, participants completed a standard dot probe task. On each trial, a fixation cross was displayed in the centre of the screen for 500 ms, followed by the presentation of a picture pair for 500 ms. The pictures were displayed on either side of the central position, with a distance of 40 mm between their inner edges. A dot probe was then displayed in the location of one of the previously presented pictures. Participants were asked to identify the location of the probe as quickly as possible, by pressing the corresponding keys labelled L (‘z’) and R (‘/’) on the computer keyboard. The dot probe remained displayed until a response was made. The inter-trial interval was 500 ms.

The task commenced with 12 practice trials, followed by 2 buffer trials and 160 experimental trials. In the experimental trials, each of the 20 critical (chocolate–non-chocolate sweet food) and 20 control (non-chocolate–non-chocolate) picture pairs was presented four times, once for each of the picture location (left, right) × probe location (left, right) combinations. Thus probes replaced each of the pictures in each pair with equal frequency (50/50). All pairs were presented in a new randomly chosen order for each participant.

#### Training

In the attentional re-training phase, participants completed a modified dot probe task. In the attend and avoid training conditions, the 20 critical (chocolate–non-chocolate sweet food) picture pairs were presented 16 times, for a total of 320 trials, with each picture appearing 8 times on each side of the screen. Attentional bias was manipulated by varying the location of the dot probes. Specifically, in the attend condition, dot probes replaced chocolate pictures on 90% of trials and non-chocolate pictures on 10% of trials, designed to direct attention toward chocolate cues. Conversely, in the avoid condition, dot probes replaced chocolate pictures on 10% of trials and non-chocolate pictures on 90% of trials, designed to direct attention away from chocolate cues. A 90–10 distribution, as opposed to a 100–0 distribution, was used to reduce the obviousness of the contingency [10].

**Fig 1:**
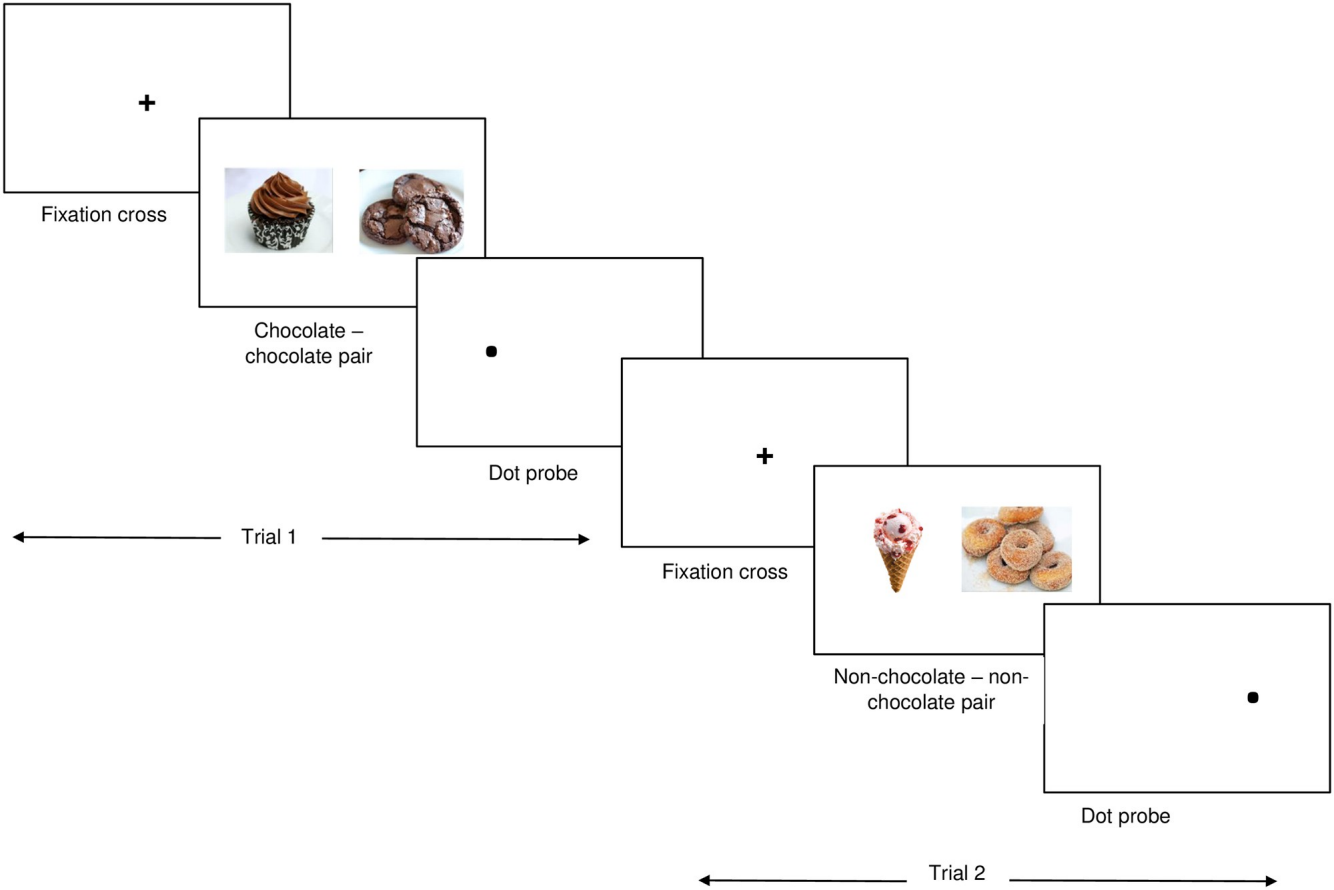
Schematic presentation of sham-n training.

In the sham-n training condition, participants were equally exposed to the same chocolate and non-chocolate sweet food items as in the attend and avoid conditions, but pairs consisted of either two chocolate pictures or two non-chocolate pictures (see Fig 1). The 10 chocolate (chocolate–chocolate) and 10 non-chocolate (non-chocolate sweet food–non-chocolate sweet food) picture pairs were presented 16 times, for a total of 320 trials, twice for each of the picture location (left, right) × probe location (left, right) combinations. Probes replaced the left and right pictures in each pair with equal frequency.

#### Post-test

The post-test was similar to the pre-test, except that there were no practice trials.

## Results

The data set is available in (S1 File).

### Statistical considerations

An alpha level of.05 was used to determine significance. Partial η^2^ was used as the effect size measure for ANOVAs; Cohen’s *d* was used for t-tests. Benchmarks for partial η^2^ are .01, small; .06, medium; .14, large; and for Cohen’s *d* .20, small; .50, medium; .80, large.

### Attentional bias

As is standard practice, data from incorrect trials (2.33%) were discarded. Additionally, response times greater than 2.5 *SD*s above or below the mean were eliminated as outliers (0.72%).

For each of the pre- and post-test phases, an attentional bias score was calculated by subtracting the mean response times to probes that replaced chocolate pictures from the mean response times to probes that replaced non-chocolate pictures on critical trials, such that a positive score indicates an attentional bias toward chocolate and a negative score indicates an attentional bias away from chocolate. These attentional bias scores were then analysed by a 3 (training condition: attend, avoid, sham-n) × 2 (time: pre-test, post-test) mixed model ANOVA.

**Fig 2:**
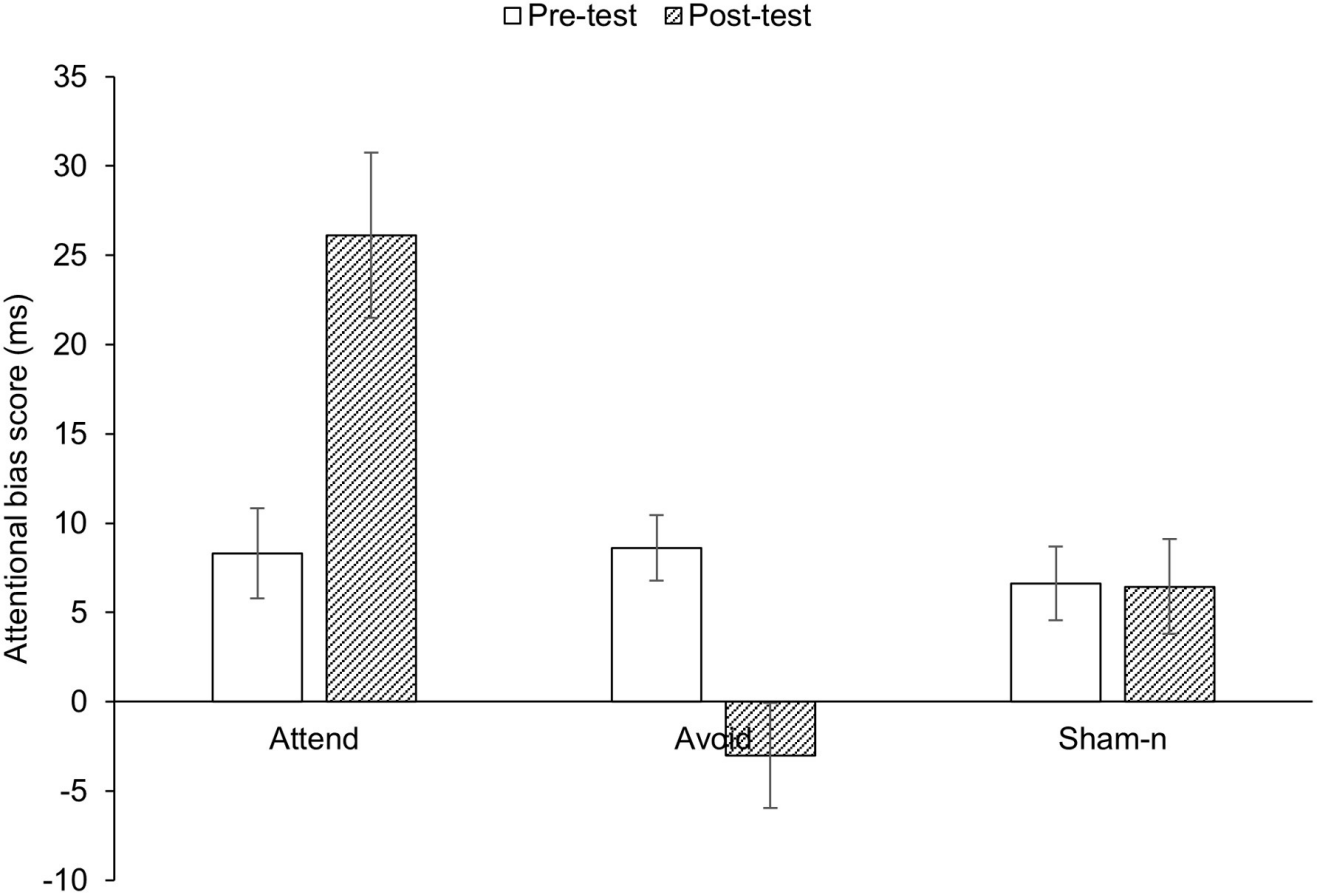
Mean attentional bias scores (with standard errors) for attend, avoid and sham-n training conditions at pre- and post-test.

The overall training condition × time interaction proved significant, *F*(2, 189) = 16.26, *p* < .001, partial η^2^ = .15. In order to separately compare the change in attentional bias in the attend and the avoid conditions against sham-n training, two 2 × 2 ANOVAs were performed. As can be seen in Fig 2, the absolute difference between pre- and post-test attentional bias scores was significantly greater for each of the attend and avoid conditions compared to the sham-n condition (attend versus sham-n: *F*(1, 126) = 10.23, *p* < .01, partial η^2^ = .08; avoid versus sham-n: and *F*(1, 126) = 6.57, *p* < .05, partial η^2^ = .05). In addition, within conditions, paired samples *t* tests showed a significant increase in attentional bias scores from pre- to post-test in the attend group, *t*(63) = 3.92, *p* < .001, *d* = .58, a significant decrease in the avoid group, *t*(63) = 3.87, *p* < .01, *d* = .58, and clearly no change in the sham-n training group, *t*(63) = .05, *p* = .961.

## Discussion

The present study provides the first empirical demonstration of sham-n training as an alternative control condition for attentional bias modification. The findings are clear. Attentional re-training was successful in that the decrease in attentional bias following avoidance training was greater than following sham-n training (as well as attend training). In addition, attentional bias for chocolate increased following the attend training, decreased following the avoidance training, and did not change following sham-n training.

The observed difference in attentional bias for chocolate following avoidance and attend training is in line with previous findings for chocolate [9, 30] and other substances including alcohol, tobacco and drugs [8, 20]. Here the reduction in attentional bias for chocolate following avoidance training was not only greater than that observed following attend training, but also greater than that following sham-n training. The present results show that both the attend training and the avoidance training altered attentional bias, but in opposite directions. More generally, the pattern of results suggests that in previous attentional bias modification studies that employed a reverse (i.e., attend) training control condition, any reported difference in attentional bias likely occurred because of a change in both directions, that is, a decrease in the avoid condition and an increase in the attend condition.

The fact that sham-n training had no effect on attentional bias is consistent with its underlying logic. Sham-n training (like sham training) matches the stimulus exposure and response requirements of attentional bias modification. But, crucially, because of its different pairing of stimuli (i.e., two substance-related stimuli *or* two neutral stimuli), there is no effect on attentional bias. By contrast, sham training has stimulus pairs that consist of one substance-related and one neutral stimulus. As probes replace the substance-related and neutral stimuli with equal frequency, over trials any pre-existing attentional bias will diminish. This unintended reduction in bias following sham training likely contributes to the lack of attentional bias modification effects found in some previous studies [11, 12, 22] and highlights the importance of including a comparison control condition in which attentional bias is not affected into the design of future attentional bias modification studies.

Like all research, the present study has some limitations. First, the findings were obtained in a sample of convenience, consisting of students who like chocolate, and thus will need to be replicated in more extreme or clinical samples such as individuals who experience uncontrollable cravings for chocolate or engage in restrictive or emotional eating. Sham-n training is likely to be particularly useful in such samples who show elevated levels of attentional bias for the desired substance. Logically, sham-n training should not change this initial bias. Second, we used the same stimulus images in all phases of the attentional bias modification protocol. Future research will need to determine whether effects generalise to new stimuli not used during the training. Third, the major limitation of the present study is that the design did not include a standard sham-training condition for comparison. Although we have demonstrated that sham-n training is inert (in contrast to standard sham training in other studies [10, 21–23]), we cannot conclude that it produces *better* results than sham training in the current protocol. An important next step will be to directly compare sham-n training with sham training. Similar control conditions to sham-n training could then be developed for protocols designed to modify other biases, such as approach bias (i.e., the tendency to reach out toward substance-related stimuli), which are also in search of an optimal control [33]. These would need to be suitably adapted to accommodate key methodological task variations such as the presentation of single stimuli.

## Conclusion

The present study used sham-n training for the first time as a comparison control condition for attentional bias modification and demonstrated it to be inert. In addition, we clearly demonstrated that avoidance training reduced attentional bias for chocolate whereas attend training increased it. Future research should investigate the usefulness of this new control condition in attentional bias modification across other appetitive domains.

## Supporting information

S1 File Study data set. (SAV)
